# Identifying reaction modules in metabolic pathways: bioinformatic deduction and experimental validation of a new putative route in purine catabolism

**DOI:** 10.1186/1752-0509-7-99

**Published:** 2013-10-05

**Authors:** Matthieu Barba, Raphaël Dutoit, Christianne Legrain, Bernard Labedan

**Affiliations:** 1 Institut de Génétique et Microbiologie, CNRS UMR 8621, Université Paris Sud, Bâtiment 400, 91405, Orsay Cedex, France; 2 Institut de Recherches Microbiologiques J.-M. Wiame IRMW, Campus CERIA, Av. E. Gryson 1, 1070, Brussels, Belgium; 3 present address: Laboratoire de Biométrie et Biologie Évolutive, CNRS UMR 5558, Université Claude Bernard Lyon 1, 69622, Villeurbanne Cedex, France; 4 present address: Bioinformatique, Laboratoire de Recherche en Informatique, CNRS UMR 8623, Université Paris Sud, Bâtiment 650, 91405, Orsay Cedex, France

**Keywords:** Dihydroorotase, Cyclic amidohydrolases, Dihydroorotase dehydrogenase, Pyrimidine metabolism, Purine metabolism, Reaction module, Functional annotation, *Rubrobacter xylanophilus*

## Abstract

**Background:**

Enzymes belonging to mechanistically diverse superfamilies often display similar catalytic mechanisms. We previously observed such an association in the case of the cyclic amidohydrolase superfamily whose members play a role in related steps of purine and pyrimidine metabolic pathways. To establish a possible link between enzyme homology and chemical similarity, we investigated further the neighbouring steps in the respective pathways.

**Results:**

We identified that successive reactions of the purine and pyrimidine pathways display similar chemistry. These mechanistically-related reactions are often catalyzed by homologous enzymes. Detection of series of similar catalysis made by succeeding enzyme families suggested some modularity in the architecture of the central metabolism. Accordingly, we introduce the concept of a reaction module to define at least two successive steps catalyzed by homologous enzymes in pathways alignable by similar chemical reactions. Applying such a concept allowed us to propose new function for misannotated paralogues. In particular, we discovered a putative ureidoglycine carbamoyltransferase (UGTCase) activity. Finally, we present experimental data supporting the conclusion that this UGTCase is likely to be involved in a new route in purine catabolism.

**Conclusions:**

Using the reaction module concept should be of great value. It will help us to trace how the primordial promiscuous enzymes were assembled progressively in functional modules, as the present pathways diverged from ancestral pathways to give birth to the present-day mechanistically diversified superfamilies. In addition, the concept allows the determination of the actual function of misannotated proteins.

## Background

Investigating the evolution of metabolic pathways requires tracing back how the enzymes that catalyze successive steps have evolved to perform specific chemical reactions [1-3]. Enzyme families are grouping all homologous gene products descending from a common ancestor by speciation and/or gene duplication. An increasingly prevailing model [4] postulates that present-day enzyme families and superfamilies are the result of the progressive divergence of ancestral proteins endowed with a promiscuous function. Contrary to the classical model proposed by Ohno [5], it is anticipated that innovation (enzyme promiscuity) preceded gene duplication and functional divergence of the paralogous copies by descent with modification [6]. To explain the appearance of many closely related families which group into mechanistically diverse superfamilies, Glasner et al. [7] have proposed to distinguish two degrees of promiscuity: shared chemistry (substrate ambiguity) and substrate binding (catalytic promiscuity). More and more data suggest that substrate ambiguity, first defined in the classical patchwork model of Jensen [8], rather than catalytic promiscuity [9], is the main road which facilitates divergence of most enzyme families [10,11].

**Figure 1:**
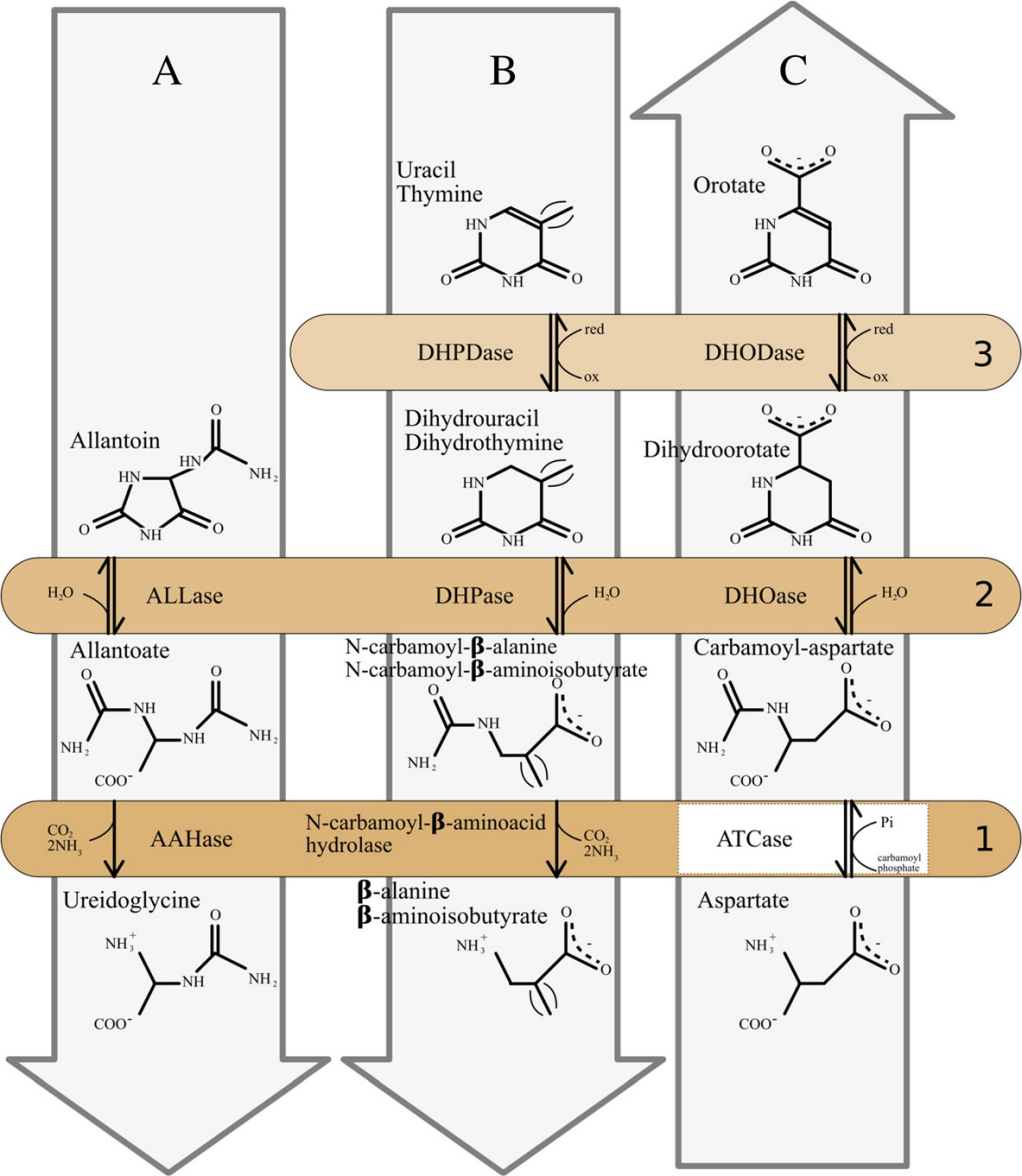
**Illustrating the respective similarities found in alignable metabolic pathways.** The chemical structures of the substrate and product of each enzyme are aligned to underline their respective similarities in the step catalyzed by the successive set of enzymes located in boxes numbered 1 to 3. The reaction modules described in the text are framed in light gray arrows labelled **A** (purine catabolism), **B** (pyrimidine catabolism), and **C** (pyrimidine anabolism). Although many reactions are reversible, the arrow orientation indicates the main direction found *in vivo*. The enzymes located in the same coloured box were found to be homologous. See list of abbreviations.

In a recent paper [12], we studied the evolutionary history of dihydroorotase (DHOase), which catalyzes the third step of pyrimidine biosynthesis, as well as that of its homologues, all members of the cyclic amidohydrolase superfamily [13,14]. We found that hydantoinase/dihydropyrimidinase, involved in degradation of pyrimidines [15], and allantoinase, a major enzyme of purine catabolism [16], are evolutionarily closer to the ancestral type of DHOase (Type I) than to the largely derived DHOases belonging to Type II and Type III. Thus, although all these homologues perform the same hydrolytic cleavage of a C-N bond in related molecules [13,14], there is no direct correlation between their respective molecular and cellular functions [12]. However, we observed that the catalyses carried out by these different homologues, defining related families which group into mechanistically diverse superfamilies, are performed on molecules displaying close chemical similarities (Figure 1, Box 2).

To examine further the observed coupling of enzyme homology and chemical similarity, we investigated the neighbouring steps in the respective pathways in purine and pyrimidine metabolism. In this paper, we identify that successive reactions display similar chemistry. These mechanistically-related reactions are often catalyzed by homologous enzymes. These homologues diverge in their molecular and cellular functions while maintaining a similar chemical mechanism in their catalytic process. This detection of series of similar catalysis made by succeeding enzyme families suggested some modularity [17,18] in the architecture of central metabolism.

This led us to propose the term *reaction module* to describe such related suites of catalyses found in parallel pathways that are alignable at the level of their chemically similar steps.

We demonstrate further the importance of this concept in the characterization of a new route in purine catabolism. After a bioinformatic discovery of a putative ureidoglycine carbamoyltransferase (UGTCase) activity, we present experimental data supporting the idea that UGTCase is likely involved in such an alternative metabolic route.

## Results and discussion

### Comparing dihydroorotate dehydrogenase and dihydropyrimidine dehydrogenase and finding new putative dehydrogenase families

Figure 1, Box 2 illustrates that DHOase, involved in pyrimidine biosynthesis (Figure 1 arrow C), is homologous both to hydantoinase/dihydropyrimidinase (HYDase/DHPase) involved in degradation of pyrimidines (Figure 1 arrow B), and to allantoinase (ALLase) a major enzyme of purine catabolism (Figure 1 arrow A). Figure 1, Box 2 underlines the similar chemical reactions performed by these different homologues on similar substrates [12]. For instance, carbamoyl-aspartate (substrate of DHOase) has a similar structure to N-carbamoyl-beta-aminoisobutyrate (the product of thymine degradation) and N-carbamoyl-beta -alanine (the product of uracil degradation). Interestingly, Figure 1, Box 3 shows further similarities in the chemical reactions carried out by the enzymes that are active in the subsequent step of pyrimidine metabolism in both anabolic (arrow C) and catabolic (arrow B) directions. Indeed, the dihydroorotate is transformed anabolically into orotate by the dihydroorotate dehydrogenase (DHODase, EC 1.3.98.1), in a process similar to the catabolic transformation (EC 1.3.1.1 and EC 1.3.1.2) of uracil or thymine to dihydrouracil or dihydrothymine by their respective dihydropyrimidine dehydrogenases (DHPDases). To improve our knowledge of the evolutionary mechanisms leading to the establishment of such related adjacent reactions (arrows B and C, Box 3), we looked further at the evolutionary relationships between DHODases and DHPDases.

**Figure 2:**
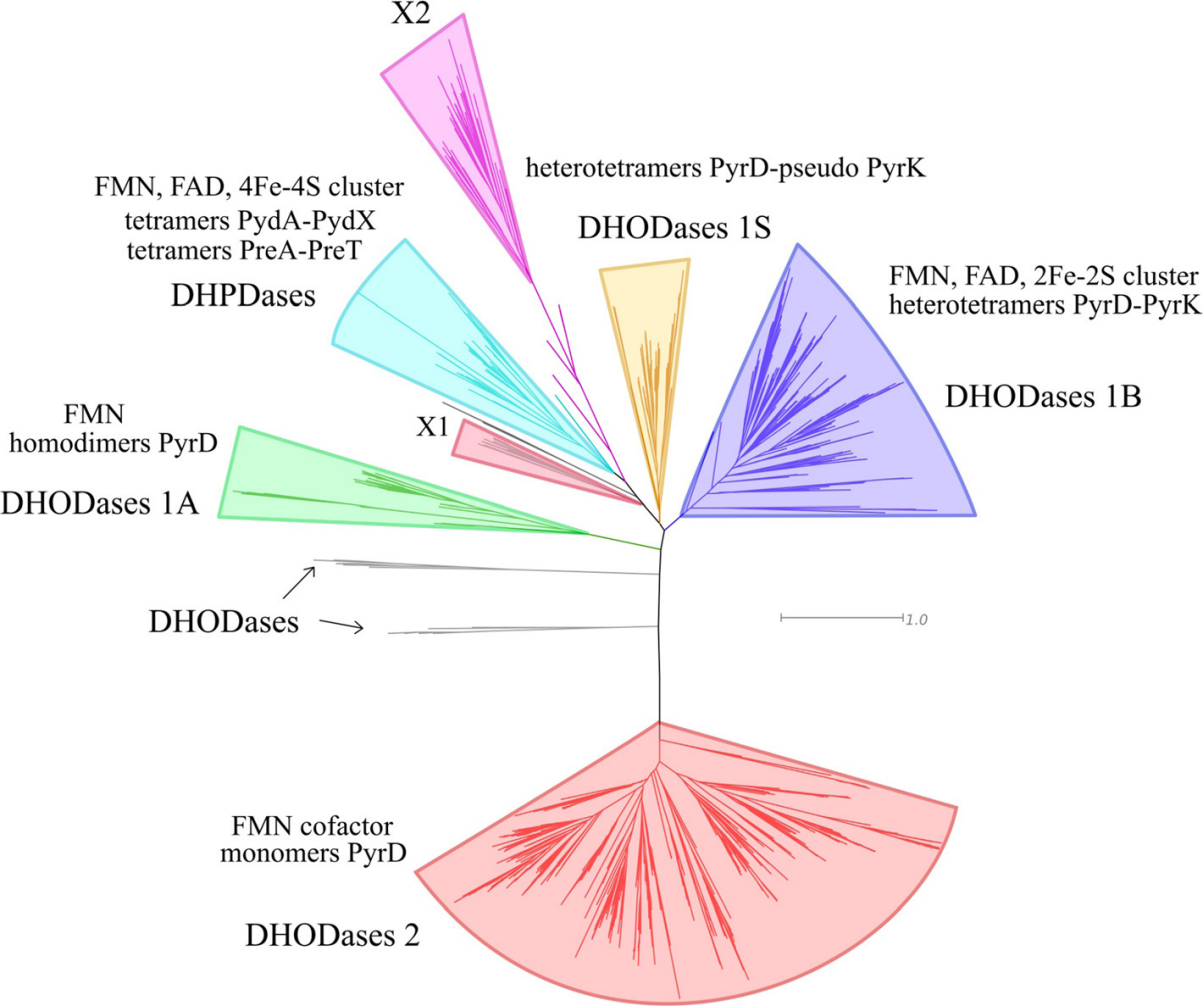
**The phylogenetic tree of the DHODases/DHPDases superfamily reveals new uncharacterized families.** This is the simplified view of the tree obtained with FastTree [58]. Complete view is available as Additional files (see below).

The methodological approach described previously by Barba et al. [12] was used to build an accurate MSA that faithfully reflects the evolutionary relationships between so many homologues displaying a large structural diversity. Moreover, the deluge of more and more varied proportions of close and distantly related amino acid sequences released by the advances in genomics makes it increasingly difficult to reconstruct an up to date phylogenetic tree. To meet these challenges, we set up a two-stage procedure summarized in Methods. First, we define a seed alignment of the amino acid sequences of PyrD (EC 1.3.98.1), PreA (EC 1.3.1.1) and PydA (EC 1.3.1.2) that have been structurally characterized. This limited set of representative sequences was build in order to be sufficiently consistent and biologically meaningful to reflect accurately the structural and functional diversity of the different families of DHODases and DHPDases. Then, as described in Methods, we added progressively to the seed alignment their homologues found in UniProtKB [19], to obtain an optimal multiple sequence alignment (MSA) of the whole superfamily (available as Additional file 1). Figure 2 shows a simplified view (the complete view is available in three different formats as Additional files 2, 3 and 4) of the topology of the phylogenetic tree obtained from this MSA, confirming that PyrD homologues are clustered in two main subtrees (each one rooting the other one). These subtrees correspond to the multimeric cytoplasmic DHODases type 1 and the monomeric membrane-bound DHODases type 2 [20]. Moreover, the sequences of DHODases 1 can be further separated into two monophyletic subclasses: the minority of PyrD subunits that form homodimers, defining a subtree containing all DHODases 1A, the majority of PyrD proteins that form heterotetramers with PyrK, defining the subtree DHODases 1B. Members of 1B subfamily share a common ancestor with four other clades: (i) the variant 1S where PyrD molecules form heterotetramers with a subunit analogous to PyrK (without obvious sequence similarity), first described in the archaeon *Sulfolobus solfataricus*[21] and found later in other Archaea; (ii) its sister subtree contains three clades, including a monophyletic group corresponding to PydA and to PreA, forming heterotetramers with PydX and PreT, respectively; (iii) diverging before these DHPDases, we found two clades of unknown dehydrogenases corresponding to newly discovered families which we provisionally call X1 and X2.

A gene coding for X2 was found in 69 bacterial species (belonging to nearly all phyla of the Domain Bacteria) as a close neighbour of a gene annotated as encoding a pyruvate-ferredoxin oxidoreductase. Moreover, in 13 out of these 69 species, the immediate neighbour to this pyruvate-ferredoxin oxidoreductase is a gene homologous to *preT*, encoding the ferredoxin part of the complex PreA-PreT of the *Escherichia coli* DHPDase [22]. In the remaining species defining the X2 subtree, this *preT*-like gene is present but is not in the same transcriptional unit as the gene for dehydrogenase X2. Since *E. coli* is found to contain four *preT* paralogues but only one copy of *preA*, one may guess by analogy that *X2* could be the partner of one of these *preT* paralogues. This should form a complex with the pyruvate-ferredoxin oxidoreductase in order to dehydrogenate an uncharacterized component that may be similar to dihydropyrimidines or hydantoin.

**Figure 3:**
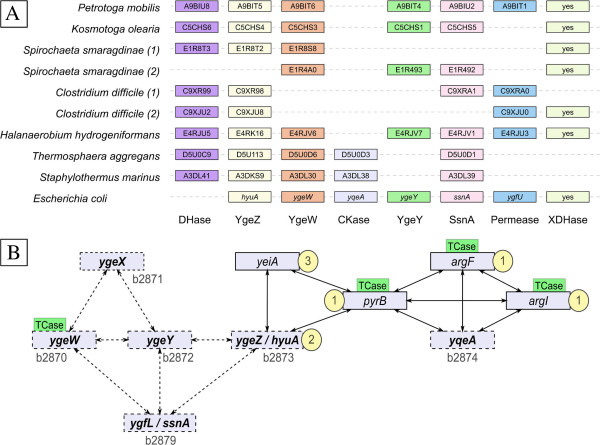
**Genomic context of genes encoding uncharacterized dehydrogenases (DHases) X1 sequences and their interactions with neighbours. A**. Genetic neighbourhoods of DHases X1 found in various organisms are schematized. The order and spacing of the genes are not respected for the sake of clarity. Except for *E. coli* where the gene name is given, the names in boxes are the UniprotKB accession number of the corresponding protein. The organisms mentioned twice with numbers (1) and (2) display two different neighbourhoods in different genome locations. “XDHase” stands for the full set of genes (*xdhA, xdhB, xdhC*) encoding the three subunits of xanthine dehydrogenase. **B**. The detected protein-protein interactions are summarized as a synthesis of individual data published by STRING database [23]. The *E. coli* gene names that are syntenic are in bold and accompanied by their Blattner identifier (b2870 and following) as published in [24]. The gene products that catalyze reactions shown in Figure 1 are highlighted by their respective box number (indicated in yellow circles). The different genes encoding a carbamoyltransferase are shown with the label TCase in green rectangles.

**Figure 4:**
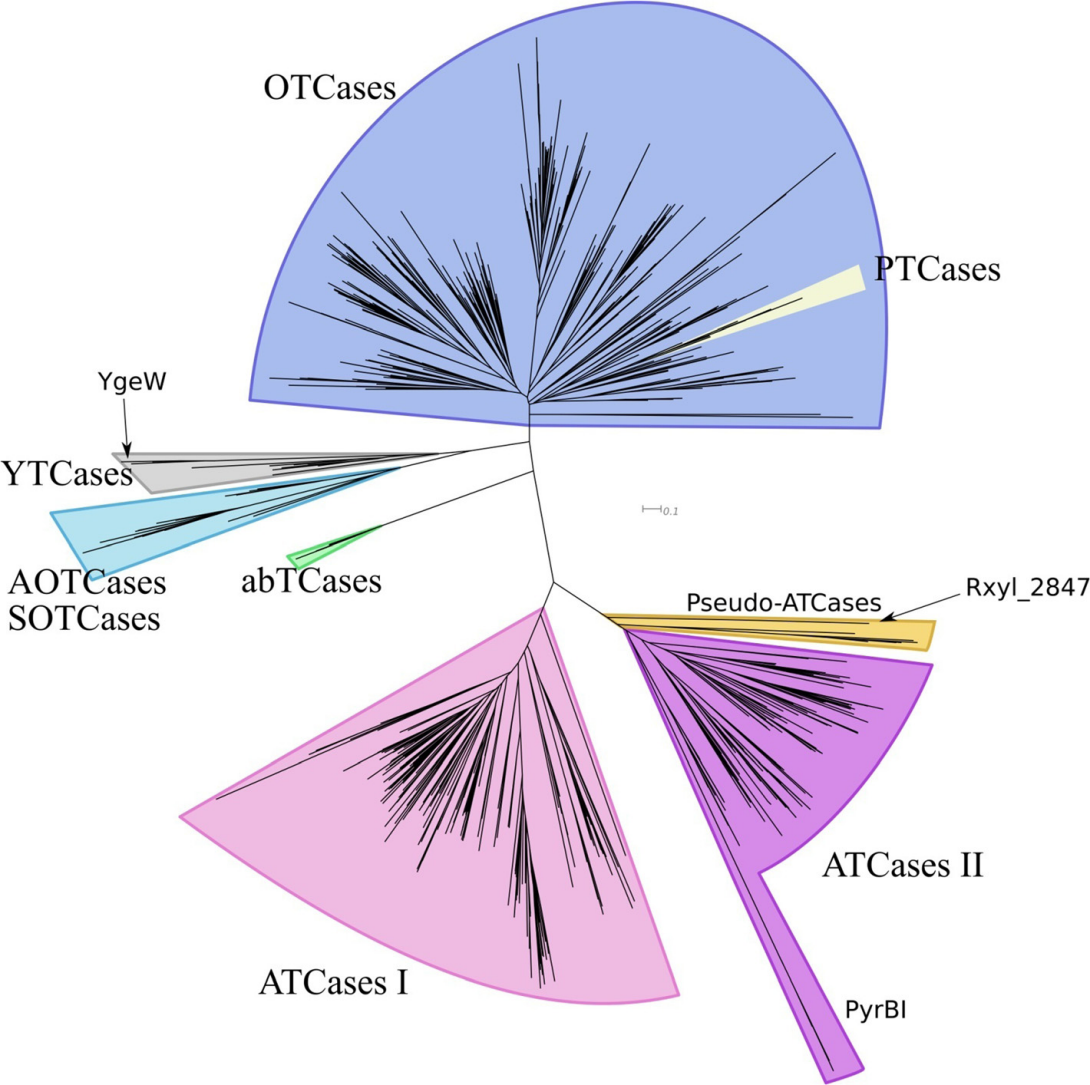
**Simplified phylogenetic tree of the carbamoyltransferase superfamily.** This is the simplified view of the tree obtained with FastTree [58]. Complete view is available as Additional files (see below).

Figure 3A summarizes the phylogenetic profile of the dehydrogenase X1 homologues and neighbouring genes in various organisms. *E. coli* is used as reference although there is – paradoxically – no DHase X1 homologue in this model organism. We found that the X1 homologue is often found together with homologues of *hyuA* (*ygeZ*) encoding a D-phenylhydantoinase (superfamily of cyclic amidohydrolases); *ygeW* encoding a hypothetical carbamoyltransferase (see below Figure 4); *ygeY* encoding a uncharacterized peptidase belonging to family M20; *ygfL* encoding an uncharacterized metal dependent aminohydrolase SsnA; *xdhA*, *xdhB*, and *xdhC* encoding the three subunits of xanthine deshydrogenase XDHase; *ygfU* encoding a xanthine/uracil permease, and, finally; *yqeA* encoding a carbamate kinase-like protein. Figure 3B further underlines, in the case of *E. coli*, that several of these gene products are known to be associated by protein-protein interactions as published in STRING database 9.05 [23] while being involved in purine salvage [24]. The gene cluster *ygeW* to *yqeA* (*b2870* to *b2874*) linked to *ygfL*/*ssnA* (*b2879*) delineate a conserved network of syntenic genes where some of the nodes (i.e., *yeiA* and *ygeZ*) are linked to genes encoding carbamoyltransferases (*pyrB* and *argF*/*argI*) and the carbamate kinase (*yqeA*). Thus, Figure 3 suggests that dehydrogenase X1 homologues are associated with conserved genes potentially involved in pyrimidine but also purine catabolism. To explore such an unexpected link between pyrimidine and purine metabolism, we inspected further the reactions described in Figure 1, Box 1. These three parallel reactions appear to be chemically similar in terms of substrate and product structures. Moreover, the allantoate amidohydrolase AAHase (arrow A) and N-carbamoyl-beta-aminoacid hydrolase (arrow B) involved in pyrimidine and purine catabolism, respectively, appear to be homologous. This is not the case, however, for the aspartate carbamoyltransferase (ATCase), which is involved in pyrimidine anabolism (Figure 1, arrow C). We thus looked for a possible undetected reaction module in the purine pathway (Figure 1, arrow A) by searching for a putative biochemical reaction that could be similar to that of the ATCase (PyrB product). Accordingly, we re-examined the phylogeny of the whole superfamily of carbamoyltransferases to look for uncharacterized homologues that could be involved in such an undetected reaction module.

### Updating the carbamoyltransferase phylogenetic tree

The methodological approach previously described [12] and summarized above and in Methods, was used to reconstruct an accurate evolutionary tree of the carbamoyltransferase superfamily. Figure 4 shows a simplified view (see Additional files 5, 6, 7 and 8 for complete views of the MSA and tree, respectively) of this updated tree. As in the trees we reconstructed previously, with far less sequences [25-27], there is a clear separation between the ornithine carbamoyltransferases (OTCases) and the ATCases (Figure 4). However, the huge increase in the number of sequences brings with it three notable features. (i) The previously described OTCase alpha and beta subfamilies appear now to be partially intermingled. (ii) The putrescine carbamoyltransferases (PTCases) form a monophyletic group that currently branches inside this OTCase subtree. This evolutionary location appears to be biologically significant since it has been recently demonstrated that the PTCase synthesized by *Listeria monocytogenes* is actually a bifunctional enzyme, catalyzing the decarbamoylation of either citrulline or carbamoylputrescine [28]. This depends on growth conditions at low pH and when expressed as a virulence factor [28]. In addition, the recent determination of the 3D structure of PTCases confirms the evolutionary inclusion of PTCases among OTCases [29,30]. (iii) The two families of ATCases (ATC I and ATC II) we described previously [25-27] still form two monophyletic subtrees corresponding to different quaternary structures [26].

**Figure 5:**
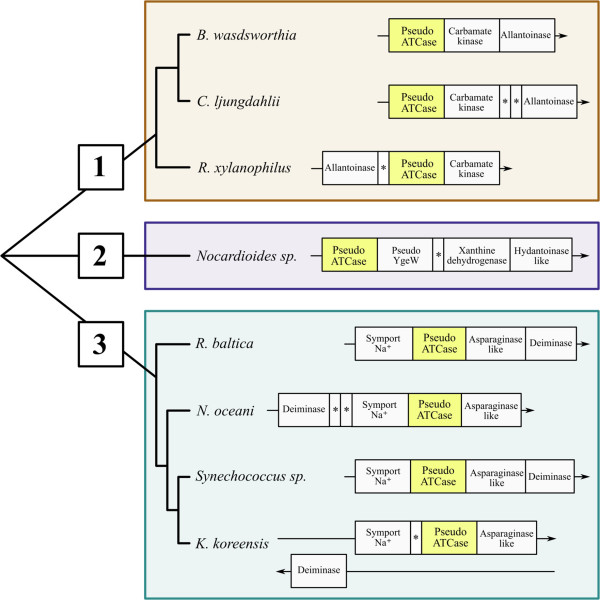
**Pseudo-ATCase subtree with its 3 subgroups and their gene contexts.** Group 1 includes *Bilophila wadsworthia* 3_1_6 (GenBank Project:PRJNA41963), *Clostridium ljungdahlii* DSM 13528 (GenBank Project: PRJNA202264) and *Rubrobacter xylanophilus* DSM 9941 (GenBank Project: PRJNA58057); group 2 includes *Nocardioides sp.* JS614 (GenBank Project: PRJNA58149); group 3 includes *Rhodopirellula baltica* SH1 (GenBank Project: PRJNA61589), *Nitrosococcus oceani* ATCC 19707 (GenBank Project: PRJNA58403), *Synechococcus sp.* WH 8102 (GenBank Project: PRJNA61581) and *Kangiella koreensis* DSM 16069 (GenBank Project: PRJNA59209). The gene encoding the pseudo-ATCase is highlighted in the yellow rectangle.

However, we now find, at the root of the ATC II subtree, a small polyphyletic subgroup which is composed of uncharacterized proteins. We call them pseudo-ATCases since these paralogues - annotated as ATCases in public databases - can be simply discriminated from the authentic ATCases found in the same organism as detailed below (see Figure 5 and Table 1). For example, in the case of *Rhodopirellula baltica*, it is easy to distinguish the gene *RB7429*, encoding a genuine ATCase (PyrB, UniProtKB: Q7UNR3), and found next to the gene *RB7430*, encoding a DHOase (PyrC, UniProtKB: Q7UNR2), from its paralogue *RB13301*, encoding the pseudo-ATCase (UniProtKB: Q7UHC6), and located in a completely different context (see Figure 5).

**Table 1:** Conserved residues among carbamoyltransferases

**Enzyme family**	**Common TCase motifs**			**ATCase motif**
	**(Positions in *Escherichia coli *** **ATCase)**			
	**1**	**2**	**3**	**4**
	**S53-T56**	**H135-Q138**	**H265-P268**	**R230-Q232**
OTCase	S[LT]RT	HPXQ	HCLP	--
AOTCase, SOTCase	S[LM]RT	HP[LC]Q	HCLP	--
YTCase	S[LT]RT	HP[TMF]Q	H[AVC]LP	--
abTCase	STRT	HPTQ	HDLP	--
ATCase (I & II)	S[TR]RT	HP[ST]Q	HP[LG]P	RXQ
**pseudo-ATCase 1**	STRT	HPTQ	**HPLA**	**AIA** or **AIS** or **SIA**
**pseudo-ATCase 2**	STRT	HPTQ	**H[ST]LP**	**GX[SC]**
**pseudo-ATCase 3**	STRT	HPTQ	**HSLP**	**VXP**

The structural motifs specific for each subgroup of pseudo-ATCases are in bold.

### Characterizing the pseudo-ATCases

Figure 5 shows that pseudo-ATCases can be differentiated into three evolutionarily-defined subgroups using their closest homologue, the *Pyrococcus abyssi* authentic ATCase, as the outgroup. Table 1 shows that this phylogenetic differentiation is also well supported by major differences, defined using the *E. coli* ATCase sequence as a reference, in the two structural motifs located in the C-terminal part (in bold). The first motif, corresponding to H265-P268 (column 3 in Table 1) distinguishes each group of pseudo-ATCases from all the other carbamoyltransferases, while the second motif R230-Q232 (column 4 in Table 1) distinguishes each group of pseudo-ATCases from the genuine ATCases. Moreover, pseudo-ATCases contain, at their N-terminal region, two structural motifs (corresponding to S53-T56 (column 1), and H135-Q138 (column 2) in the *E. coli* ATCase sequence) that are highly conserved in the whole superfamily as a carbamoyltransferase signature.

Additionally, the phylogenetic differentiation of each of the three subgroups of pseudo-ATCases is confirmed by a distinctive gene context (Figure 5). In Subgroup 2 (composed of only one organism, the actinobacterium *Nocardioides sp*. JS614), the gene encoding the pseudo-ATCase is adjacent to a homologue of the *E. coli ygeW* gene. YgeW [31] is a carbamoyltransferase which belongs to a group sharing a common ancestor with the AOTCases [32] and SOTCases [33] (see Figure 4 and Table 1), but its true physiological role remains elusive [31]. In *Nocardioides,* the adjacent downstream genes are *xdhA, xdhB* and *xdhC*, together encoding a putative heterotrimeric xanthine dehydrogenase (involved in purine degradation [24]), and then a gene encoding a putative phenylhydantoinase HyuA [16]. Such gene association is reminiscent of the specific context of X1 family (Figure 3) even if there is no detectable X1 homologue in *Nocardioides sp*. JS614.

Members of the Subgroup 3 pseudo-ATCases are found in marine bacterial species. The encoding gene is part of a conserved syntenic block containing on one side a gene encoding a Na^+^ symporter (seawater milieu?) and on the other side a gene encoding a putative asparaginase. In nearly all cases, this cluster is adjacent to a gene annotated as encoding a putative deiminase, which is most probably an N-carbamoyl-L-amino acid amidohydrolase (HyuC) involved in hydantoin metabolism [34].

The three species defining Subgroup 1 exhibit a block of genes directly involved in purine metabolism, namely a carbamate kinase and an allantoinase, next to the pseudo-ATCase (Figure 5). Moreover, the gene context of *Rubrobacter xylanophilus* pseudo-ATCase *Rxyl_2847* (UniProtKB Q1AS69) is particularly intriguing since it includes a gene cluster composed of several operons involved in purine degradation. The operon encompassing genes *Rxyl_2840* to *Rxyl_2850* (Table 2) is implicated in successive steps of degradation to allantoate. Xanthine dehydrogenase genes (*Rxyl_2836* to *Rxyl_2839*) are found upstream of this operon, while genes involved in the degradation of glyoxylate to D-glycerate (last steps of purine catabolism) are located downstream in a third transcription unit (*Rxyl_2851* to *Rxyl_2854*).

**Table 2:** **Genomic context of the *Rubrobacter xylanophilus *** **pseudo-ATCase**

**Gene id**	**Uniprot AC**	**Uniprot annotation**	**Proposed annotation**
*Rxyl_2840*	Q1AS76	uracil/xanthine permease	uracil/xanthine permease
*Rxyl_2841*	Q1AS75	uncharacterized protein	OHCU decarboxylase
*Rxyl_2842*	Q1AS74	CMP/dCMP deaminase,	CMP/dCMP deaminase
*Rxyl_2843*	Q1AS73	uricase	uricase
*Rxyl_2844*	Q1AS72	5-hydroxyisourate hydrolase	5HIU hydrolase
*Rxyl_2845*	Q1AS71	allantoinase	allantoinase
*Rxyl_2846*	Q1AS70	uncharacterized protein	Unknown
*Rxyl_2847*	Q1AS69	ATCase	**see text**
*Rxyl_2848*	Q1AS68	carbamate kinase	carbamate kinase
*Rxyl_2849*	Q1AS67	asparaginase	**see text**
*Rxyl_2850*	Q1AS66	transcriptional regulator	transcriptional regulator

UniprotKB data are as published in UniProt release 2013_05 (May 1, 2013). The annotations proposed for genes Rxyl_2847 and Rxyl_2849 are detailed in the text (see text in bold).

### Deducing a novel carbamoyltransferase activity in a reaction module involved in purine degradation

The degradation process of allantoin to glyoxylate may involve one of several possible enzymes, namely, allantoicase, allantoate amidohydrolase, ureidoglycine amidohydrolase, ureidoglycolatase, and ureidoglycolate amidohydrolase [35-38]. Importantly, however, we could not detect in the *R. xylanophilus* genome (RefSeq: NC_008148) any gene encoding the catalytic step corresponding to any of these enzymes. This suggested the possibility that *Rxyl_2847* and its neighbours (Table 2) could play a role in this pathway. Accordingly, we searched for possible reaction modules composed of chemically similar substrates/products through the comparison of purine catabolism (Figure 1A), pyrimidine catabolism (Figure 1B), and pyrimidine biosynthesis (Figure 1C).

**Figure 6:**
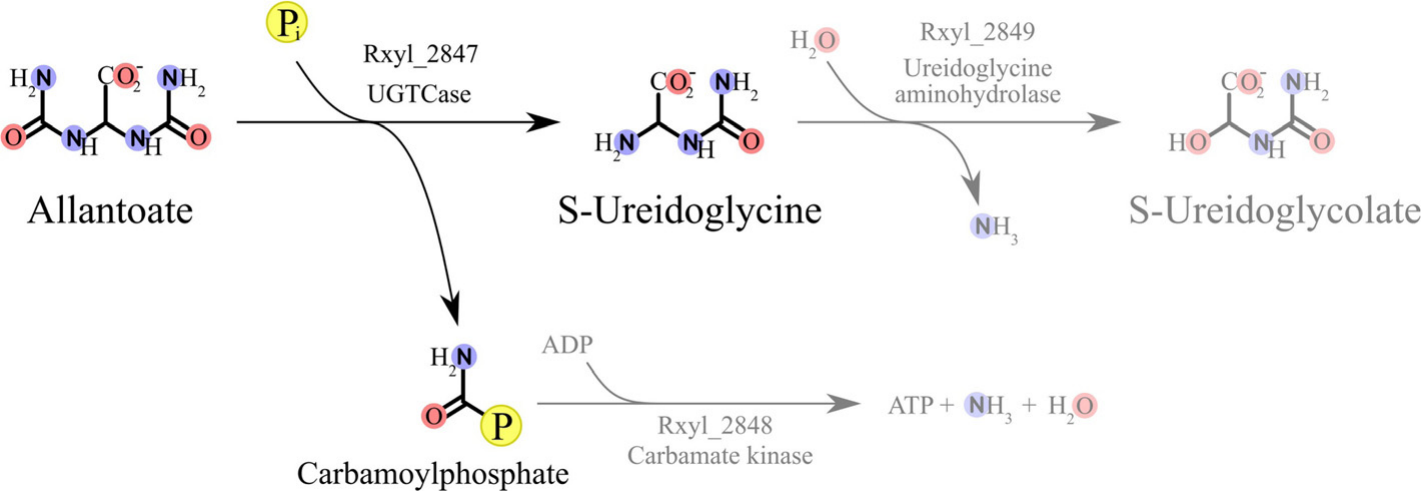
**Proposed purine degradation pathway in *Rubrobacter xylanophilus.*** The proposed reaction degrading allantoate to S-ureidoglycine and carbamoyl phosphate is in black. The reactions that are presumed to be catalyzed by the neighbouring genes (*Rxyl_2848* and *Rxyl_2849*) are written in light gray. The encircled P stands for phosphate, P_i_ for inorganic phosphate.

Figure 1, Box 1 shows the functional similarities of ATCase with the pyrimidine catabolic N-carbamoyl-L-amino acid amidohydrolase (deiminase) and the purine catabolic allantoate amidohydrolase (AAHase) [39]. This suggests that the product of the *Rxyl_2847* gene may play a role as a carbamoyltransferase to functionally replace the AAHase. In addition, *Rxyl_2847* is followed by *Rxyl_2848*, a gene annotated as encoding a carbamate kinase (Figure 5). Consequently, we propose to reclassify the pseudo-ATCase Rxyl_2847 as an ureidoglycine carbamoyltransferase (UGTCase), which would catalyze the reaction: allantoate + P_i_ < = > ureidoglycine + carbamoyl-phosphate in *R. xylanophilus* (Figure 6).

### Indirect evidence that Rxyl_2847 has a ureidoglycine carbamoyltransferase activity

**Figure 7:**
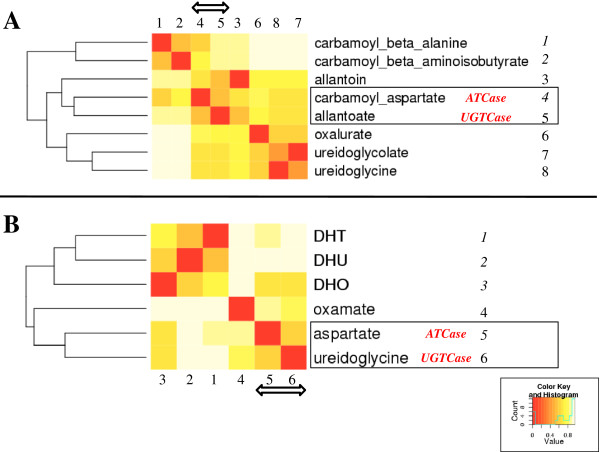
**ChemMine dendrograms and heatmaps.** Similarity between carbamoylated **(A)** and uncarbamoylated **(B)** compounds that are known or potential substrates of carbamoyltransferases is shown as dendrograms and heatmaps as computed using ChemMine [40]. DHT = dihydrothymine; DHU = dihydrouracil; DHO = dihydroorotate. Count, is the number of compound-compound comparison in each value range from 0 to 1. Value, is the Tanimoto coefficient minus 1 for each comparison [40].

Although a reaction module transforming allantoate to ureidoglycine appeared to be the most logical reaction that we are looking for, we could not exclude the alternative possibility of a promiscuous carbamoyltransferase activity normally involved in purine degradation, being responsible for transformation of allantoate to glyoxylate. To address this point, we quantified the chemical similarity of all the potential substrates and products of Rxyl_2847 with that of ATCase, i.e., aspartate and carbamoyl-aspartate (Figure 1C Box 1), using ChemMine [40]. Figure 7A shows the dendrogram obtained using the Tanimoto coefficient (see Methods) to compare all potential carbamoylated substrates from purine catabolism (allantoin, allantoate, ureidoglycine, ureidoglycolate and oxalurate), as well as those from pyrimidine catabolism (carbamoyl-β-alanine, carbamoyl-β-aminoisobutyrate), with pyrimidine anabolism (carbamoyl-aspartate). Likewise, a second dendrogram (Figure 7B) was obtained by comparing the same decarbamoylated counterparts as potential products (dihydrothymine (DHT), dihydrouracil (DHU), dihydroorotate (DHO), oxamate, aspartate, and ureidoglycine). Both dendrograms and their corresponding deduced heatmaps show that among all possible compounds usable as respective substrate/product couples of the predicted UGTCase, allantoate (Figure 7A) and ureidoglycine (Figure 7B) are the most similar to aspartate and carbamoyl-aspartate (the substrate/product couple of ATCase). These similarities are underlined by a double arrow in the heatmaps and framed in the dendrograms (Figure 7A and Figure 7B). Therefore, it becomes chemically legitimate, in the case of *R. xylanophilus*, to substitute the AAHase molecular function (Figure 1A Box 1) by that of the predicted UGTCase (Figure 6) in order to perform the cellular function transforming allantoate into ureidoglycine. Noticeably, such a chemical closeness of Rxyl_2847 with ATCase supports the phylogenetic proximity and sequence similarity of the suggested UGTCase with genuine ATCases in the frame of our reaction module concept.

### Experimental validation that Rxyl_2847 has a ureidoglycine carbamoyltransferase activity

To ascertain the bioinformatic deduction that Rxyl_2847 is really an UGTCase, the *Rxyl_2847* gene was cloned into a pBAD expression vector (see Methods and Additional file 9). Unexpectedly, the sequencing of plasmid pCEC53 revealed several mismatches between the cloned ORF sequence and the published genomic sequence of *R. xylanophilus* DSM9941 (RefSeq: NC_008148). An extended DNA fragment encompassing the ORF *Rxyl_2847* was generated by two independent PCR reactions, sequenced, and this confirmed the sequence of the cloned *Rxyl_2847* (GenBank : JX289826).

**Figure 8:**
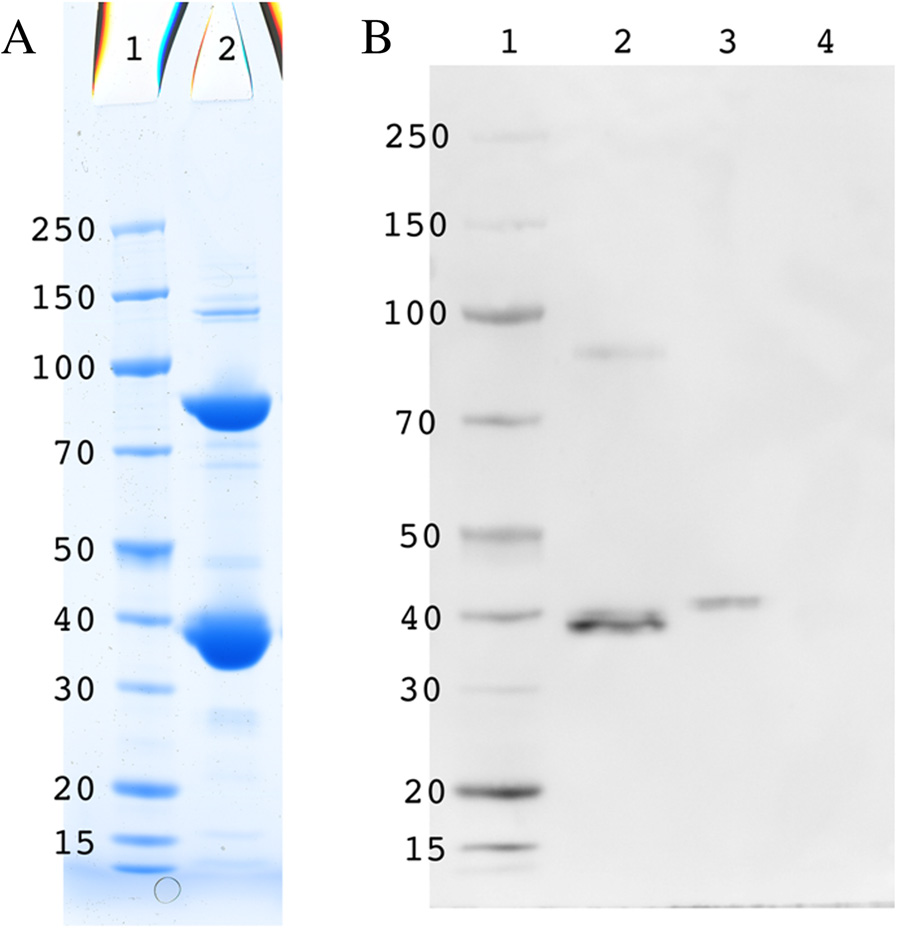
**Analysis of the purified Rxyl_2847 enzyme. (A)** SDS-PAGE of purified Rxyl_2847 enzyme on a NuPage Bis-Tris 4-12% gel (Life Technologies) in MOPS running buffer. Proteins were stained with PageRuler protein staining solution (ThermoScientific). Lane 1, PageRuler Unstained Broad Range Protein Ladder (ThermoScientific); Lane 2, 10 μg of purified Rxyl_2847 enzyme. **(B)** Western blot analysis of purified Rxyl_2847 enzyme (same condition of electrophoresis as in **(A)**, electroblot onto Hybond (GE Healthcare) nitrocellulose membrane). Lane 1, PageRuler Unstained Broad Range Protein Ladder (ThermoScientific); Lane 2, 100 ng of purified Rxyl_2847 enzyme; Lane 3, 100 ng of a purified His-tagged protein control; Lane 4, 100 ng of a purified untagged protein control.

Recombinant His-tagged enzyme was purified to near-homogeneity by a three-step procedure including heat-treatment, metal affinity chromatography and molecular sieving (see Figure 8 and Methods). SDS-PAGE showed a subunit molecular mass of 37 kDa but also a major band at 80 kDa (Figure 8A). The western blot analysis of purified enzyme (Figure 8B) pointed out that it corresponds to a dimeric state of Rxyl_2847. Such phenomenon was already reported for other thermophilic enzymes [41,42]. The activity of the purified enzyme was examined in the physiological, catabolic direction, i.e. the phosphorolysis of allantoate. Since the equilibrium of the reaction catalyzed by carbamoyltransferases strongly favours the carbamoylation direction, *in vitro* studies of the catabolic reaction require the removal of one of the products formed. This can be achieved by using arsenate instead of phosphate [43] or by coupling the reaction *in vivo* to that of a carbamate kinase, or an anabolic carbamoyltransferase. In this work, the *E. coli* OTCase, purified as described previously [44] was used in the presence of ornithine to convert the carbamoyl phosphate produced by the phosphorolysis of allantoate to citrulline (Table 3).

**Table 3:** **Characterization of the *Rubrobacter xylanophilus *** **UGTCase activity**

**Reaction mixture composition**	**Assay temperature**	**Specific activity **^**c **^**(μmol min**^**-1**^ **mg**^**-1**^**)**
25 mM potassium arsenate pH 6.8, 20 mM allantoate	37°C	0.046 ± 0.007 ^a^
25 mM Hepes pH 6.8, 20 mM allantoate	37°C	n.d. ^a^
50 mM potassium phosphate pH 6.8, 20 mM allantoate, 5 mM ornithine, EcOTCase (100 units)	37°C	0.050 ± 0.002 ^b^
25 mM Hepes pH 6.8, 20 mM allantoate, 5 mM ornithine, EcOTCase (100 units)	37°C	n.d. ^b^
50 mM potassium phosphate pH 6.8, 5 mM ornithine, EcOTCase (100 units)	37°C	n.d. ^b^
50 mM potassium phosphate pH 6.8, 20 mM allantoate, 5 mM ornithine, EcOTCase (100 units)	60°C	5.913 ± 0.584 ^b^
50 mM potassium phosphate pH 6.8, 5 mM ornithine, EcOTCase (100 units)	60°C	n.d. ^b^

^a^assessed by quantifying ureidoglycine.

^b^assessed by quantifying citrulline.

^c^Data are the mean ± SD from at least three independent experiments. n.d., not detectable as defined in Methods.

To assay if the purified recombinant protein had a carbamoyltransferase activity, we first examined its ability to catalyze the arsenolytic cleavage of allantoate (Table 3). Importantly, since ureidoglycine, the putative product of the reaction, is thermally unstable, assays were performed with an incubation time not exceeding 5 min. (see Methods for details). The amino content in the reaction mixture was immediately analyzed by reverse phase HPLC after pre-column derivatization with o-phtaldialdehyde (see Methods). One prominent peak, corresponding to ureidoglycine (retention time: 11.6 min), was obtained after the enzyme was incubated at 37°C with allantoate and arsenate. After incubation at 60°C (the optimal growth temperature of *R. xylanophilus*[45]), only a small amount of ureidoglycine was observed, probably due to the lability of this product. Ureidoglycine formation was observed when UGTCase was incubated with allantoate and arsenate, but not in the absence of arsenate, excluding the possibility of enzymatic hydrolysis of allantoate (Table 3).

The physiological reaction catalyzed by UGTCase, namely the phosphorolysis of allantoate, was then analyzed by coupling with purified *E. coli* OTCase to prove that carbamoyl phosphate was effectively formed in the reaction. The citrulline produced in the coupled assay was quantified by reverse phase HPLC after pre-column derivatization with o-phtaldialdehyde. Table 3 demonstrates that carbamoyl phosphate was indeed produced. Comparison of the activities at both 37° and at 60°C showed that UGTCase was much more active at 60°C, as expected for a thermophilic enzyme (Table 3).

## Conclusion

In this paper, we have substantiated the potency of the concept of the reaction module to unravel undisclosed functional relationships in central metabolism and to discover the actual function of misannotated proteins [46,47], especially when coupled with an informative gene context. The so-called pseudo-ATCases (Figure 4) were found to be such an example of uncharacterized paralogues when we demonstrated they are unrelated to authentic ATCases (Figure 5 and Table 1). Using our conceptual approach, we have deduced and experimentally proved that the Rxyl_2847 protein, the pseudo-ATCase detected in the extremophile *R. xylanophilus*[45], is an UGTCase involved in the degradation of allantoin to ureidoglycine (Figure 6).

By analogy with steps observed in other species [37,48,49], we further suggest that this reaction is part of a new route of purine catabolism, where ureidoglycine is then degraded by the carbamate kinase Rxyl_2848 to produce carbamoyl-phosphate (Figure 6). Moreover, spontaneous degradation of ureidoglycine at 60°C (growth temperature of *R. xylanophilus*) would provide eventually glyoxylate (as well as ammonia and urea). We propose that *Rxyl_2849*, adjacent to *Rxyl_2847* and *Rxyl_2848*, also plays a crucial role in this newly described pathway (Figure 6). In fact, Rxyl_2849 has been annotated as an asparaginase-like enzyme in the *R. xylanophilus* genome (RefSeq: NC_008148). In contrast, Gravenmade *et al*. [50] claimed that allantoate amidohydrolase isolated from *Streptococcus allantoicus* could hydrolyze allantoate to ureidoglycolate with the release of CO_2_ and NH_3_^+^. In this case, AAHase is expected to produce ureidoglycine, which is later transformed into ureidoglycolate by an ureidoglycine aminohydrolase. Thus, in *R. xylanophilus*, Rxyl_2849 may also catalyze this deamination of ureidoglycine to ureidoglycolate, as the logical step following the action of the UGTCase Rxyl_2847 (Figure 6)*.* Although this prediction remains to be experimentally validated, it would introduce another category of reaction module. In that case, while substrates and products are chemically similar, the enzymes catalyzing analogous reactions could be evolutionarily unrelated. Consequently, automated detection of such modules with the currently available bioinformatic tools would be less simple than in the case of enzyme homology. However, it remains possible when using tools like ChemMine [40] to compare various substrates and products to detect similar chemical functions in alignable pathways.

Use of the reaction module concept should be of great value when studying mechanistically diversified superfamilies [7,11,13,14,46,47]. Reaction modules could be viewed as the elementary bricks used to assemble functional modules [17,18]. More generally, defining progressively these different elements will help to trace how the present pathways progressively diverged from ancestral pathways where the successive primordial enzymes [4] were promiscuous and gave birth to the present-day superfamilies.

## Methods

### Building a reference multiple sequence alignment (MSA) of superfamilies

We developed a two-step approach to obtain an MSA reflecting the structural and functional diversity of enzyme superfamilies. In a first step, we collected the limited set of homologues that have been both crystallized (published in the Protein Data Bank [51]) and experimentally studied, as indicated in UniProtKB/SwissProt [19].These sequences were multiply aligned using the Expresso update of the 3D-Coffee program [52] that has been benchmarked as optimal when sequence identity between target and template falls below 50% [53]. The automated alignment was further improved by hand to define a seed MSA. In a second step, an HMM profile of this seed was created to screen UniProtKB using HMMsearch [54]. This allows the identification of suitable (threshold of E-value = 10E-15) homologues that were further clustered using Cd-hit [55]. For each cluster, an automated MSA was built with MUSCLE [56] and an HMM profile (HMM_cluster) was computed. In parallel, another HMM profile was computed for the closest homologous sequences present in the seed alignment (HMM_seed). Then, the two profiles, HMM_cluster and HMM_seed, were aligned using the HHalign program [57]. A stepwise approach allows progressive addition of each aligned cluster to the seed alignment. To make this step-up more efficient and safer, we started with highly matching sequences (at least 70% identity), and the whole process was repeated while the identity threshold was progressively decreased 60, 55, 50, 45, and 40%. This allowed us to exclude a few unreliable distant sequences and to assort the individual tribes that are part of each aligned cluster.

A script was designed to detect the emergence of new homologues each time a new version of UniProtKB [19] was published. These presumptive homologues were assessed and added to the reference alignment using the HMM stepwise approach described above. Accordingly, we worked at any one time with a reliable reference MSA that was always up to date.

### Reconstructing phylogenetic trees

Seed and reference MSA were used to derive phylogenetic trees with approximate maximum likelihood approaches (FastTree version 2.1 [58]). Robustness of the reconstructed tree topologies was assessed using a bootstrap approach or a much faster alternative, the approximate likelihood-ratio test (aLRT [59]). The trees obtained (written in Newick format) were visualized using MEGA 5.1 [60] or Dendroscope 3.2.2 [61] programs.

### Functional annotation by monophyly

With the deluge of new genome sequences, phylogenetic trees contain more and more functionally unknown sequences branching together with a few experimentally characterized proteins. We used topological information of elementary subtrees to annotate uncharacterized leaves as follows. When two monophyletic subtrees, sharing a common ancestor, each contain at least one of their leaves with the same experimentally assessed functional annotation, then this function is transferred to their whole subtree, on the assumption that this shared feature comes from their common ancestor. If this is not the case, each monophyletic subtree is considered independently, tentatively divided in two more elementary subtrees and the analysis continued until the most distal subtrees coincide with leaves. Such a cautious approach prevents the introduction of damaging overinterpretation of functional proximity.

### Chemical and structural comparison of potential substrates

We used ChemMine tools [40] to compare systematically potential substrates and products of putative enzymes. Their hierarchical clustering was calculated by all-against-all comparisons of chemically related compounds using atom pair similarity measures. We used the Tanimoto coefficient, which is defined as c/(a + b + c), where c is the number of features common in both compounds, while a and b are the number of features that are unique in one or the other compound, respectively. For each cluster, the similarity scores generated were transformed into distance values, allowing creation of a dendrogram and then a heatmap that highlights the hierarchical clustering of the analyzed compounds.

### Cloning and heterologous expression of *Rxyl_2847*

*Rubrobacter xylanophilus* strain DSM9941 obtained from Deutsche Sammlung von Mikroorganismen und Zellkulturen GmbH (DSMZ) was grown aerobically at 60°C on a rotary shaker in complex medium (9 g of tryptic soy broth, 4 g of yeast extract, 3 g of NaCl, H_2_O to 1 L, adjusted to pH 7.5). Genomic DNA was extracted according to Magarvey *et al*. [62] and we used Pfu DNA polymerase (ThermoScientific) to amplify the open reading frame (ORF) *Rxyl_2847* with primers ocej475 (5’- tttaactttaagaaggagatatacatacccatgcagaaagaggcggtaaggga -3’) and ocej476 (5’- atccgccaaaacagccaagctggagaccgtctaatgatgatgatgatgatgcgcccccacgatagcggcgac -3’). The PCR product was inserted into the pBAD vector (Life Technologies) by homologous recombination in *E. coli* MC1061 [63] after growth on LB broth in the presence of 100 μg/mL ampicillin. The resulting pCEC53 plasmid was verified by sequencing (Genetic Service Facility, University of Antwerp, Belgium). For additional sequencing of the ORF *Rxyl_2847*, a PCR fragment extending from nucleotide 2853037 to nucleotide 2854149 of the published genome of *R. xylanophilus* DSM9941 (accession number GI:108764099) was generated with Pfu DNA polymerase and primers ocej483 (5’- ctcttcgagaaggcctgagaatag -3’) and ocej484 (5’- tcgtcctttatgagggagttgc- 3’). The PCR product was cloned subsequently with the CloneJet PCR cloning kit (ThermoScientific) and sequenced.

### Production and purification of recombinant Rxyl_2847 protein

*E. coli* MC1061 transformed with the expression vector pCEC53 was grown at 37°C in two litres of LB broth supplemented with 100 μg/mL ampicillin. Expression of *Rxyl_2847* was induced in mid-exponential phase by adding 0.2% arabinose, followed by overnight growth at 18°C. Cells were harvested by centrifugation, suspended in 50 ml 0.05 M potassium phosphate buffer pH 7.5, containing Complete EDTA-free protease inhibitor cocktail (Roche Applied Science) and disrupted by sonication (Ultrasonic Inc., W-225R). Insoluble particles were pelleted at 17,500 g for 30 min (Sorvall RC-6, SS34 rotor). The cell extract was heated at 60°C for 15 min and coagulated proteins were removed by centrifugation for 30 min at 17,500 g. The cleared lysate was submitted to ion metal affinity chromatography on Ni-nitrilotriacetic acid agarose resin (Qiagen) in 0.05 M potassium phosphate buffer, pH 7.5, containing 0.3 M NaCl. Elution was performed in three steps with increasing concentrations (0.1, 0.25, and 0.5 M) of imidazole. Fractions corresponding to the elution peak at 0.25 M imidazole were pooled and applied to a Superdex 200 (GE Healthcare, 16/70 column) gel filtration resin in 0.02 M Hepes buffer, pH 7.5, containing 0.15 M NaCl and 10% glycerol. Fractions containing the protein of interest were pooled and concentrated using Vivaspin 15R 30 kDa (Sartorius) membranes. The presence and purity of the recombinant enzyme was checked throughout the purification procedure by SDS-PAGE and its identity verified by Western blot. Western blot analysis was carried out as previously described [41], Rxyl_2847 enzyme was detected using PentaHis antibodies (Qiagen) and Amersham ECL Prime western blotting reagents (GE Healthcare).

### Enzymatic synthesis of ureidoglycine

Ureidoglycine is not commercially available and was generated by enzymatic hydrolysis of allantoate, catalyzed by purified recombinant *E. coli* allantoate amidohydrolase as referred to in French and Ealick [48]. Plasmid EcCD00311947 carrying the *E. coli allC* gene under the control of a T7 promoter was obtained from DNASU Plasmid Repository (The Biodesign Institute/ Arizona State University, USA). *E. coli* strain BL21(DE3) was transformed with this plasmid and grown in LB broth supplemented with 50 μg/mL kanamycin to allow expression of recombinant *E. coli* AAHase with a 6xHis tag fused to its N-terminus. Expression of AAHase was induced in mid-exponential growth phase by adding 1 mM IPTG, followed by growth for 4 h at 37°C. All purification steps were performed as described above except that the thermal treatment was omitted.

### Enzymatic assays

Enzyme activities were measured in 200-μl assay mixtures whose composition is detailed in the Results section. After incubation, the reaction was stopped by freezing on ice and the enzyme was removed from the reaction mixture by ultrafiltration on a Vivaspin 500 3 kDa (Sartorius) membrane. The products of the reaction were immediately analyzed by reverse phase HPLC after pre-column derivatization with o-phtaldialdehyde. The fluorescent derivatives of amino compounds were prepared according to Hill *et al*. [64] and analyzed by reverse phase HPLC on an Alltech Altima C18 5 μm column (150/4.6) as referred to in Jones *et al.*[65]. Initial conditions were 75% solvent A (tetrahydrofuran - methanol - 0.05 M sodium acetate (1:19:80) pH 5.9), 25% solvent B (methanol - 0.05 M sodium acetate (80:20) pH 5.9). The gradient program (flow rate of 1 ml min-1) was as follows: 75% solvent A + 25% solvent B for 1 min from the initiation step of the program; linear step to 80% solvent B in 14 min; isocratic step at 80% solvent B for 3 min; linear step to 100% solvent B for 7 min; isocratic step to 100% solvent B for 5 min. One unit of activity is defined as the amount of enzyme that converts 1 μmol of substrate to product per min under the assay conditions. Specific activity is defined in units per mg protein and activity was considered as not detectable when less than 0.001 μmol per min per mg. Protein concentration was determined by measurement of the UV absorbance at 280 nm and by the Bradford method, with bovine serum albumin as the standard.

## Abbreviations

DHOase: Dihydroorotase; ALLase: Allantoinase; DHPase: Dihydropyrimidinase; HYDase: Hydantoinase; DHODase: Dihydroorotate dehydrogenase; DHPDase: Dihydropyrimidine dehydrogenase; XDHase: Xanthine deshydrogenase; TCase: Carbamoyltransferase; ATCase: Aspartate carbamoyltransferase; OTCase: Ornithine carbamoyltransferase; PTCase: Putrescine carbamoyltransferase; AAHase: Allantoate amidohydrolase; AOTCase: Acetylornithine carbamoyltransferase; SOTCase: Succinylornithine carbamoyltransferase; UGTCase: Ureidoglycine carbamoyltransferase; MSA: Multiple sequence alignment; HMM: Hidden Markov model.

## Competing interests

The authors declare that they have no competing interests.

## Authors’ contributions

MB conceptualized the reaction module idea, carried out the sequence alignment and phylogeny studies, and performed all the bioinformatics approaches to deduce the new carbamoyltransferase activity. RD and CL carried out the molecular biology and enzymology necessary to obtain the experimental demonstration of the newly discovered carbamoyltransferase activity. BL conceived the study, participated in its design and coordination and drafted the manuscript which was further improved (and approved) by all authors.

## Supplementary Material

Additional file 1 This is the complete MSA of DHases in FASTA format. Click here for file

Additional file 2 **Complete tree of DHases can be viewed in three different formats (Newick **[60]**, NeXML http://www.nexml.org/, and Dendroscope **[61]**).** Click here for file

Additional file 3 **Complete tree of DHases can be viewed in three different formats (Newick **[60]**, NeXML http://www.nexml.org/, and Dendroscope **[61]**).** Click here for file

Additional file 4 **Complete tree of DHases can be viewed in three different formats (Newick **[60]**, NeXML http://www.nexml.org/, and Dendroscope **[61]**).** Click here for file

Additional file 5 This is the complete MSA of carbamoyltransferases in FASTA format. Click here for file

Additional file 6 **Complete tree of carbamoyltransferases can be viewed in three different formats (Newick **[60]**, NeXML http://www.nexml.org/, and Dendroscope **[61]**).** Click here for file

Additional file 7 **Complete tree of carbamoyltransferases can be viewed in three different formats (Newick **[60]**, NeXML http://www.nexml.org/** ***, and Dendroscope *** [61]**). ** Click here for file

Additional file 8 **Complete tree of carbamoyltransferases can be viewed in three different formats (Newick **[60]**, NeXML http://www.nexml.org/, and Dendroscope **[61]**).** Click here for file

Additional file 9 **Analysis of the PCR-amplification of *Rxyl_2847 *** **gene by agarose gel electrophoresis.** Click here for file
