# Supplementary figures and images for: Identifying reaction modules in metabolic pathways: bioinformatic deduction and experimental validation of a new putative route in purine catabolism
Source: BMC Syst Biol. 2013 Oct 5;7:99. doi: 10.1186/1752-0509-7-99 (PMC4016543; doi:10.1186/1752-0509-7-99)

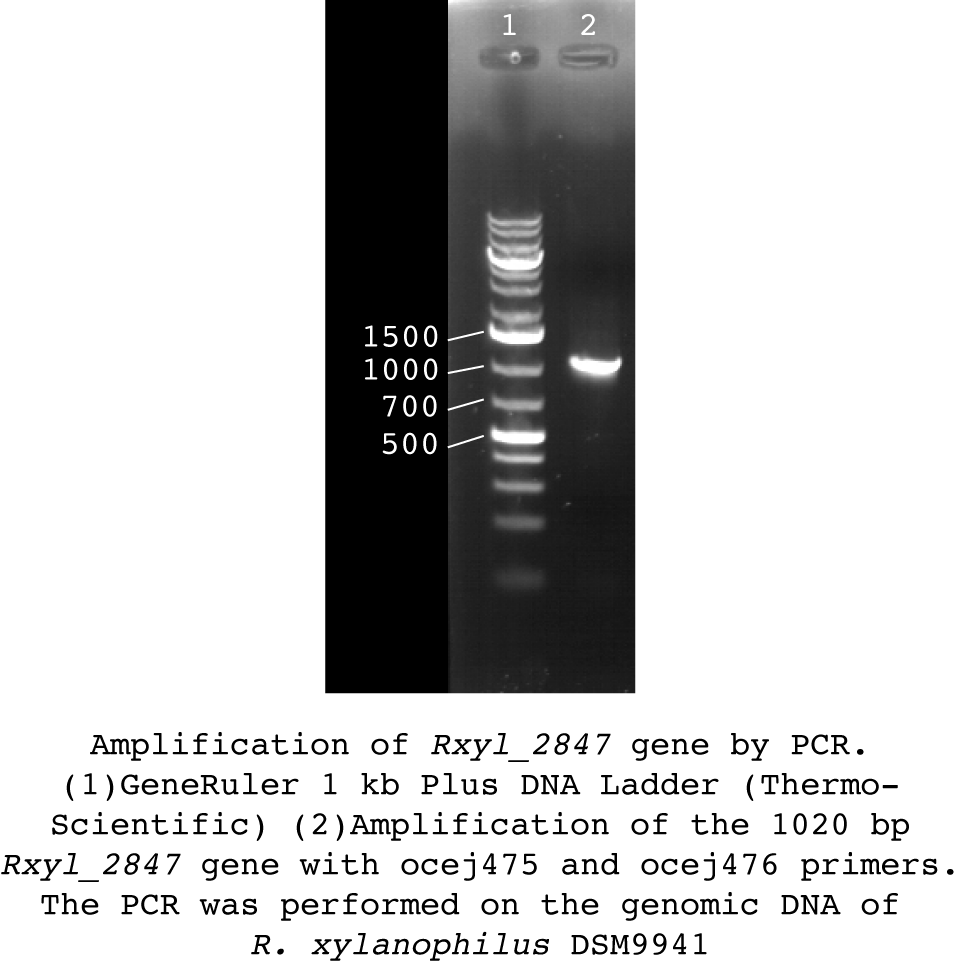

Supplement: Additional file 9 — Analysis of the PCR-amplification of Rxyl_2847 gene by agarose gel electrophoresis. [file 1752-0509-7-99-S9.tiff]
